# Depressive Symptom Change Patterns during the COVID-19 Pandemic and Their Impact on Psychiatric Treatment Seeking: A 24-Month Observational Study of the Adult Population

**DOI:** 10.1155/2024/1272738

**Published:** 2024-08-05

**Authors:** Omid V. Ebrahimi, René Freichel, Sverre Urnes Johnson, Asle Hoffart, Ole André Solbakken, Daniel J. Bauer

**Affiliations:** ^1^Department of Experimental Psychology, University of Oxford, Oxford, UK; ^2^Department of Psychology, University of Oslo, Oslo, Norway; ^3^Department of Psychology, University of Amsterdam, Amsterdam, Netherlands; ^4^Modum Bad Psychiatric Hospital and Research Center, Vikersund, Norway; ^5^Department of Psychology and Neuroscience, The University of North Carolina at Chapel Hill, Chapel Hill, USA

## Abstract

Despite the presence of individual differences in the depressive symptom change in adults during the COVID-19 pandemic, most studies have investigated population-level changes in depression during the first year of the pandemic. This longitudinal repeated-measurement study obtained 39,259 observations from 4,361 adults assessed nine times over a 24-month period in Norway (March 2020 to March 2022). Using a Latent Change Score Mixture Model to investigate differential change patterns in depressive symptoms, five profiles were identified. Most adults revealed a consistently resilient (42.52%) or predominantly resilient pattern differentiated by an initial shock in symptomatology (13.17%). Another group exhibited consistently high depressive adversities (8.5%). One group showed mild deterioration with small increases in depressive symptomatology compared to onset levels (29.04%), and a second strong deterioration group exhibited clinically severe levels of gained symptoms over time (6.77%). Both deteriorating depressive symptom change patterns predicted the presence of a psychiatric diagnosis and treatment seeking at the end of the study period. Together, the absence of a preexisting psychiatric diagnosis at the onset of the pandemic and severe symptom increases during, combined with reports of psychiatric treatment seeking and diagnosis at the end of the study period, indicated that the strongly deteriorating subgroup represents an additional and newly emerged group of adults struggling with depressive problems. Factors related to general adverse change (lower education levels, lone residence), initial shocks prior to recovery (frequent information seeking, financial and occupational concerns), and resilience and recovery (older age, being in a relationship, physical activity) were identified. Binge drinking and belonging to an ethnic minority were influential predictors of the strongly deteriorating group. All major change patterns in depressive symptoms occurred during the first 3 months of the pandemic, suggesting this period represents a window of sensitivity for the development of long-lasting depressive states versus patterns of recovery and resilience. These findings call for increased vigilance of psychiatric symptoms during the initial phases of infectious disease outbreaks and highlight a specific target period for the implementation of preventive measures.

## 1. Introduction

The global pandemic caused by the coronavirus disease (COVID-19) strained essential domains in society, including the economy, health, and healthcare systems, with changes in average population-level mental health being reported [1, 2]. Among the psychiatric symptom domains most strongly tied to the pandemic stands depression [2, 3]. While there is evidence for heterogeneity in the depressive symptom change patterns of adults, most studies have investigated overall population-level fluctuations in depressive symptomatology [4, 5]. Such investigations lack the ability to disaggregate differential patterns in the evolution of depressive symptoms during the pandemic [6].

Results from the early pandemic stages indicate that certain groups in society were disproportionately affected by the COVID-19 health crisis [7, 8, 9]. Alongside resilient response patterns to mental health adversities, initial evidence suggests that a group of individuals showed an early worsening in mental health that was sustained through the first year of the pandemic [6, 10, 11, 12]. These early findings add to the evidence that different depressive symptom change patterns may be present in the adult population [5].

To date, however, little research has been conducted to evaluate population heterogeneity in depressive symptom change beyond the first year of the pandemic [11, 12], with more research needed on the risk factors predicting long-term differences in depressive symptom expression [4, 13, 14]. Beside demographic descriptors, risk factors on the population level that could be related to differences in depressive symptom change patterns include increases in alcohol intake as a potential coping mechanism [15]; news consumption, related to the mass dissemination of pandemic-related information and individual differences with news engagement [16]; ethnicity, to investigate impact of the pandemic in minority groups [17]; job and financial concerns related to the pandemics' economic repercussions [18]; and physical activity as a potential protective factor against symptom development [19].

Notably, a key gap in the literature concerns a need to understand future adverse outcomes related to differential depressive change patterns during the pandemic [1, 4]. That is, it is unclear whether different depressive symptom change patterns during the pandemic period relate to adverse future clinical outcomes beyond symptomatology, including treatment seeking and diagnosis [1, 4].

Leveraging nine longitudinal assessment waves, the present study seeks to address these gaps by investigating differential depressive symptom change patterns in adults over a 24-month period. Factors tied to resilient and adverse depressive change patterns in periods of infectious disease will be investigated. Finally, the extent to which depressive change profiles can predict adverse future outcomes is examined by investigating whether different symptom change profiles occurring during the first 2 years of the pandemic can predict psychiatric diagnosis and treatment-seeking behavior at the end of this period. This extends the literature by moving beyond the experience of symptoms and investigating the impact of symptom change patterns on future adverse clinical outcomes.

## 2. Materials and Methods

### 2.1. Study Design and Participants

This study is part of The Norwegian COVID-19, Mental Health and Adherence Project (MAP-19), ethically approved by The Regional Committee for Medical and Health Research Ethics (reference: 125510). MAP-19 is a large-scale longitudinal study designed to investigate depressive symptomatology in the general adult population across the pandemic period. The duration of the study was 24 months, covering the full containment-accompanying (i.e., mitigation protocol implemented) pandemic period, from the onset of these protocols to their termination in Norway (i.e., March 2020 to March 2022). Eligible participants were all adults (i.e., including and above 18 years old) who resided in Norway across the assessment period, providing informed consent to partake in the study.

### 2.2. Procedures

The sampling procedure was designed to recruit a proportionate number of subjects from each region of the country with respect to the region's size. Upon recruitment in March 2020 (T1), subjects responded to an online survey disseminated to a random selection of Norwegian adults using a Facebook algorithm, in addition to systematic dissemination of the survey via national, regional, and local information platforms (i.e., television, radio, and newspapers). This procedure is elaborated in detail elsewhere [4, 20]. The final assessment of the study was conducted in March 2022, resulting in nine overall repeated measures of the adult population.

### 2.3. Stratification of Sample and Quality Control of Data

The demographic features of the sampled subjects were contrasted with their known prevalence rates in the population. Available attributes not fully representative of the adult population were poststratified to be proportional to their known rate, harmonizing parameters in the sample to the population parameter to render sample characteristics as close to the target adult population as possible. The final stratified sample included 4,361 adults (T1: Assessment period: March to April, 2020), with the coverage at each wave, including 2,158 (T2: June to July, 2020), 2,239 (T3: November to December, 2020), 1,963 (T4: January to February, 2021), 1,811 (T5: May 2021), 1,405 (T6: July to August 2021), 1,426 (T7: October to November, 2021), 1,110 (T8: January 2022), and 1,269 (T9: March to April 2022) participants. Attrition levels were consistent with other longitudinal studies during the pandemic [6]. A tree-based machine learning classification approach was used to inspect attrition in the study, with no systematic patterns of attrition found in the data [4]. The quality of the data was further assessed using attention checks [21]. This was examined through the following item being added to the survey, where participants were asked to “Please provide the response “A little” if you are paying attention to this survey,” including the following response options (1: Not at all; 2: A little; 3: Moderately; 4: A lot; 5: Extremely). 97.80% of the participants passed the attention check, and subjects failing the check were excluded to assure high data quality.

### 2.4. Measurement

#### 2.4.1. Sociodemographic Characteristics

Respondents provided demographic information, including their age (18–30 years; 31–44 years; 45–64 years; and 65 years and above), biological sex, relationship status (single; in a relationship), education level (compulsory school; upper secondary high school; student; any university degree), living status (lives with others; lives alone), history of psychiatric disorders (no; yes), and ethnic minority status (no; yes). Additionally, contextual risk and protective factors, including physical activity frequency, binge drinking (no; yes), frequency of information seeking about the pandemic (0: Not at all to 7: Multiple times per hour), and financial and occupational concerns were reported (0: Not at all to 6: Almost every day).

#### 2.4.2. Depressive Symptomatology

Depressive symptoms were assessed with the Patient Health Questionnaire (PHQ-9; [22]), consisting of nine items covering the DSM symptom criteria of depression [23] measured on a four-point Likert-scale (0–3; 0: Not at all, 3: Nearly every day). Sum scores range from 0 to 27, with higher scores indicating greater depression severity. Scores greater than 10 have been found to be indicative of depressive diagnosis with moderate severity with a sensitivity and specificity of 88% [22]. Scores below 5 reveal no sign of depression and no clinical relevance. The questionnaire was formally translated and found to have sound psychometric properties [24] and shown to be appropriate for longitudinal investigation (cf. measurement invariance) of depression in the Norwegian population and the present sample [4]. The scale further revealed excellent internal consistency across the full study period (Cronbach's *α* of 0.88 at T1 and ranging from 0.90 to 0.92 at T2 to T9).

#### 2.4.3. Psychiatric Diagnosis and Treatment Seeking

The presence of a psychiatric diagnosis was assessed at the onset of the study (i.e., to control for preexisting mental health conditions) and at the end of the study period. This was measured by asking the subjects whether they had received a psychiatric diagnosis as assessed and provided by a healthcare professional. In order to identify the presence of a newly emerged psychiatric diagnosis during the pandemic period, subjects who endorsed this item at the end of the study (T9; March 2022) and who had not reported the presence of any preexisting psychiatric diagnosis previously were classified as endorsing the presence of a novel psychiatric condition at T9, 2 years into the pandemic (March 2022). Such self-report measures have been found to be valid for measuring mental disorders (e.g., [25, 26]).

In addition to the measurement of depressive symptoms (PHQ-9) and the presence of a psychiatric diagnosis, subjects were asked to report on their treatment-seeking behavior at the end of the study period (T9; March 2022). This was measured by querying subjects about whether they were receiving psychiatric treatment for their experienced mental health problems [4].

### 2.5. Statistical Analyses

**Figure 1:**
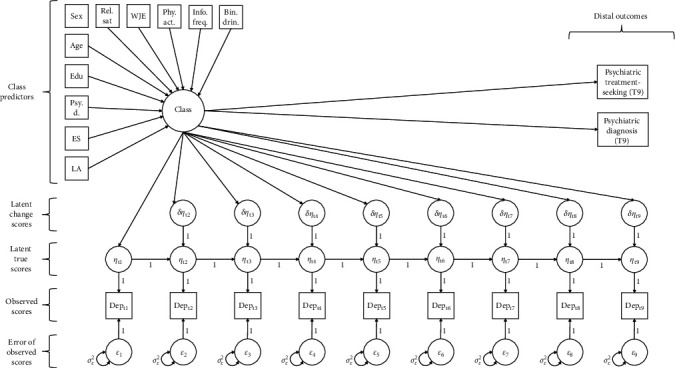
The latent change score mixture model (LCSMM) along with the demographic and contextual predictors of class membership. The covariance between *η*_t1_ and the latent change scores *δη*_t2−t9_, the estimated parameter labels of the class predictors, covariances between class predictors, and the regression estimates from the class predictors to *η*_t1_ and *δη*_t2−t9_ are omitted from the figure for visualization purposes. All estimated parameters are class-specific, and the subscript “_C_” was omitted to enhance visualization. Within-class variance is restricted to zero to obtain latent classes, with all variability in latent change captured by between-class differences. Bin. drin., binge drinking; Edu, education; ES, ethnic status; Info. freq., information seeking frequency; LA, living alone; Psy. d., psychiatric diagnosis; Rel. stat., relationship status; Phy. act., physical activity; WJE, worry about job and economy.

All statistical analyses were performed using Mplus (Version 8.3) and R (Version 4.3.1). Change profiles in depressive symptomatology were captured via a latent change score model (LCSM; [27]), enabling the estimation of nonlinear symptom change patterns during the pandemic. Model fit for the LCSM was determined by root mean square error of approximation (RMSEA) ≤ 0.05, comparative fit index (CFI) ≥ 0.95, Tucker–Lewis Index (TLI) ≥ 0.95, and standardized root mean squared residual (SRMR) ≤ 0.05 [28]. After computing the average profile of change in symptomatology across the 2-year period for the overall population, the presence of distinctive depressive symptom change patterns across subgroups (i.e., latent classes) was investigated by extending the model into a latent change score mixture model (LCSMM). Whereas the standard LCSM assumes graded, continuous differences in latent change parameters across individuals in the population, the LCSMM instead semi-parametrically captures individual differences via discrete latent classes that reflect prototypical change profiles. Unique advantages of the LCSMM for this investigation include that: (1) it inherits from the standard LCSM the ability to capture nonlinear profiles of true change and to recover these with less bias and greater power than the analysis of observed difference scores contaminated by measurement error [29]; and (2) it allows for the estimation of distinct patterns of change over time, characterizing differences in the evolution of depressive symptoms during the pandemic. A path diagram detailing the specification of the LCSMM is provided in Figure 1. Each of these models were estimated by maximum likelihood (ML) using the full dataset, including records with partial missing data. The treatment of missing data by ML is considered state-of-the-art [30]. This enables the inclusion of all available data in the analysis, allowing the retention of records with partial missing data as opposed to the practice of dropping such records in complete-case analysis. The ML approach thus decreases bias and increases statistical power relative to complete-case analysis [30].

Following best practices for latent class models, class enumeration was based on consideration of multiple statistical and substantive criteria [31, 32, 33, 34, 35]. First, to ensure the recovery of robust and meaningful classes, a minimum class size of 5% was set, and only models for which the solution and log-likelihood could be replicated across random initializations were considered [31, 34]. Second, information criteria (IC) values were compared, which seek to balance model fit versus complexity between models with different numbers of classes [35]. In practice, with very large samples like the one analyzed here, ICs often continue to decrease, sometimes trivially, as more classes are added to the model. Accordingly, scree plots were used to identify when drops in IC values gave way to small improvements, that is, the point of diminishing returns, such that the selected number of classes would capture the principal patterns present in the data without becoming trained on less important fine detail (“splitting hairs”) or chance sampling variation [34]. Third, models with high-class separation (measured by entropy) were favored [32, 34], indicating higher distinctiveness of the latent classes and less ambiguity in identifying covariates related to class membership and predicting adverse future outcomes by class membership. Last, these statistical criteria were cross-checked with substantive considerations [34], inspecting model solutions to ensure that new classes captured clinically meaningful differences in level and/or change and favoring class solutions consistent with previous findings in the literature. Full details of the class enumeration procedure are outlined in the *Supplementary 1*.

Following class enumeration, classes were further characterized by considering their relations to external variables, including both class predictors and distal outcomes. To identify factors predictive of differential depressive symptom change patterns, the ML-based 3-step procedure of Vermunt was implemented [36], providing a multinomial regression of class membership on each of the aforementioned demographic characteristics and contextual risk and protective factors. Additionally, the predictive utility of the latent classes for adverse outcomes at the final wave of the study (T9) was examined, including psychiatric treatment seeking and reported psychiatric diagnosis, using the 3-step procedure of Bolck et al. [37] as extended by Vermunt [36]. These 3-step approaches [38] obviate the potential for class distortion (redefinition of the classes) with the introduction of external variables while accounting for the uncertainty of class membership to mitigate bias due to classification error.

## 3. Results

### 3.1. Participant Characteristics

The baseline demographic characteristics of the participants are presented in Table 1. Overall, 49.34% of the sample were female (compared to 49.96% in the population), and 35.38% had a university degree (versus 35.60% in the population). Fewer individuals with an ethnic minority background were in the sample (5.16% versus 16.80%), and the sample was younger compared to the Norwegian population (mean age: 37.48 versus 48.84). The prevalence of preexisting mental health conditions in this sample was 19.49%, representative of the known rate of psychiatric disorders in the Norwegian adult population between 16.66% and 25.00% [39]. The quota of adults sampled from each region of the country was further proportional to the respective region size, providing a geographically representative sample of Norway.

Participant characteristics were predominantly stable over time (see *Supplementary 1*). Across the 2-year study period, slightly more single individuals (*p* < 0.05) and slightly more individuals above 65 years (*p* < 0.05) compared to younger individuals remained in the study. These differences in retention and dropout rates were, however, negligible, with a 1.07% dropout in single individuals and 2.36% dropout in individuals between 18 and 30 years from T1 to T9. No other demographic characteristic showed significant differences in retention or dropout rates over time (*p* > 0.05). Overall, this highlighted that no specific subgroups revealed influentially disproportional attrition rates over time across the study period.

### 3.2. Model Fit and Class Selection

The LCSM estimated population-level change and yielded excellent fit to the data, with *χ*^2^ (29) = 110.53 (*p*  < 0.001), RMSEA = 0.025 (90% CI: [0.020, 0.030]), CFI = 0.992, TLI = 0.991, and SRMR = 0.033. The figure in *Supplementary 2* shows the aggregated population-level change patterns in depressive symptoms across the study period. This overall pattern masked within-population heterogeneity, with comparison of LCSMMs ranging from one to eight classes leading to the selection of the five-class model as the optimal of the different depressive symptom change patterns (*Supplementary 1*). Models with more classes consistently produced lower IC values, however, diminishing returns were observed after five classes. Additionally, the five-class model produced a stable solution, with replication of the highest log-likelihood across multiple random initializations, whereas models with more classes evidenced instability (i.e., the highest log-likelihood could not be replicated across initializations). The five-class model also yielded the highest entropy (0.71), with these classes exceeding 5% of the population and class profiles capturing the principal patterns of change within the data. Five classes were further is consistent with previous results during the early phase of the pandemic [6, 12]. Accordingly, the five-class model was selected to provide a stable and substantively meaningful representation of the data that optimally balanced parsimony and fit.

### 3.3. Profiles of Change across the Pandemic Period

**Figure 2:**
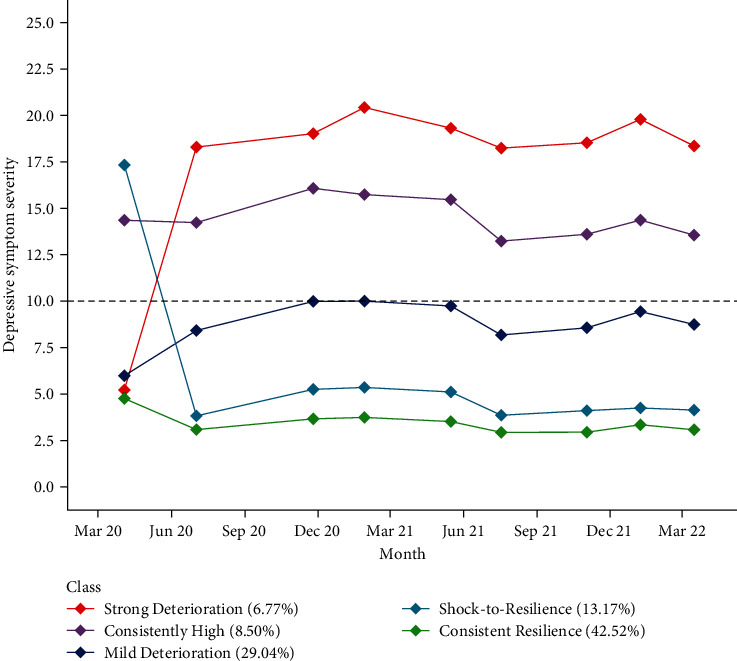
Differential depressive symptom change patterns across the 24-month study period. The dashed line presents the validated cutoff for moderate levels of depression.

Five distinctive change patterns in depressive symptom expression were identified across the 2-year pandemic period.  Table 2 presents the estimated initial levels and latent changes over time of the five change patterns in depressive symptoms.  Figure 2 shows the longitudinal profiles captured by these estimates. The individual change profiles for a random subset of 100 adults in each of the five (i.e., posterior probability ≥ 0.9 of belonging to the respective profile) are further provided in *Supplementary 3*.

Two profiles were characterized by resilience. A large subgroup representing 42.52% of adults displayed consistently low depressive symptoms throughout the pandemic (i.e., Consistent Resilience class), following a slight elevation at the start of the pandemic that resolved to consistently low levels throughout the remainder of the study period. Another class, encompassing of 13.17% of the adult population, exhibited an initial shock in symptomatology at the onset of the pandemic but likewise recovered swiftly and displayed stably low levels of depressive symptoms from the third month of the pandemic and forward (Shock-to-Resilience class).

Two subgroups of adults revealed deteriorating change patterns in depressive symptoms occurring during the first 3 months of the pandemic. One subgroup (Mild Deterioration class), consisting of 29.04% of the adult population, exhibited modest increases in symptom levels across the first year of the pandemic, with the largest increase occurring during the first 3 months of the pandemic, followed by slight decreases in symptom levels during the second year. This group showed a mean increase of 2.77 in depressive symptoms at the end of the study period (March, 2022; mean depression scores: 8.76) compared to at the onset of the pandemic period (March, 2022; 5.99). The other subgroup consisting of 6.77% of the adult population (Strong Deterioration class) revealed a pronounced pattern of major deterioration in depressive symptom expression, exhibiting a critical change in depressive symptomatology in moving from a predominantly asymptomatic level to symptom levels indicative of a moderate-to-severe depressive state during the first 3 months of the pandemic (*δη*_t2_; Table 2). This strong deterioration group showed a mean increase of 12.14 in depressive symptoms by the end of the 2-year study period (mean: 17.36) compared to the onset of the pandemic period (mean: 5.22).

Finally, a last class emerged, encompassing about 8.50% of adults who revealed consistently high levels of depressive symptoms during the pandemic period (Consistently High class).

### 3.4. Predictors of Class Membership

Several key predictors of class membership were identified. In all analyses, the largest class, Consistent Resilience, was used as the reference category for comparison.  Table 3 displays the increase or decrease in odds associated with being in each of the other classes relative to the Consistent Resilience class (i.e., the odds ratios).

Compared to Consistent Resilience, the odds of Strong Deterioration were significantly higher for those living alone (OR 2.98 [95% CI: 1.65–5.40]), those who started binge drinking during the pandemic (OR 16.78 [2.86–98.52]), and those identifying as an ethnic minority (OR 3.66 [1.36–9.81]). Higher education levels were associated with lower odds of Strong Deterioration relative to Consistent Resilience (OR 0.78 [0.60–0.99]).

The odds of Mild Deterioration relative to Consistent Resilience were higher among individuals residing alone (OR 2.36 [1.46–3.81]), binge drinkers (OR 6.84 [1.17–39.88]), and those engaging in more frequent information seeking (OR 1.22 [1.01–1.47]). Higher age (OR 0.69 [0.56–0.84]) and increases in physical activity (OR 0.84 [0.73–0.97]) served as protective factors that reduced the odds of Mild Deterioration versus Consistent Resilience.

The odds of Consistently High symptom levels relative to the Consistent Resilience were higher for those having a preexisting psychiatric diagnosis prior to the pandemic period (OR 12.21 [6.02–24.76]), binge drinking (OR 13.99 [2.26–85.92]), living alone (OR 3.13 [1.52–6.43]), and displaying higher financial and occupational concerns (OR 1.51 [1.26–1.80] per unit increase). Greater physical activity (OR 0.59 [0.46–0.77]), being in a relationship (OR 0.51 [0.27–0.97]), and older age (OR 0.43 [0.29–0.65]) reduced the odds of being in the Consistently High class compared to Consistent Resilience.

Finally, the odds of Shock-to-Resilience relative to Consistent Resilience were higher among those who engaged in information seeking about the pandemic more intensively (OR 1.30 [1.06–1.56]), resided alone (OR 2.03 [1.14–3.59]), had a preexisting diagnosis (OR 11.35 [6.57–19.61]), and financial and occupational worries (OR 1.53 [1.35–1.74]); and lower among older adults (OR 0.45 [0.34–0.60]), those in a relationship (OR 0.52 [0.32–0.83]), with higher education levels (OR 0.73 [0.58–0.92]), and higher levels of physical activity (OR 0.67 [0.57–0.79]).

### 3.5. Class Membership and Adverse Future Outcomes

**Figure 3:**
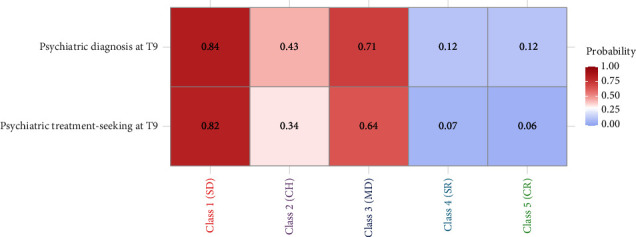
Probability of psychiatric treatment-seeking behavior and psychiatric diagnosis for adults with different depressive symptom change patterns at the end of the 2-year study period (T9). CH, Consistently High (Class 2); CR, Consistent Resilience (Class 5); MD, Mild Deterioration (Class 3); SD, Strong Deterioration (Class 1); SR, Shock-to-Resilience (Class 4).

The identified depressive symptom change patterns were used to predict two important clinical outcomes at the final assessment of the study, psychiatric treatment seeking, and reported psychiatric diagnosis, 2 years after the commencement of the identified change patterns.  Figure 3, displaying the probability of revealing these two adverse outcomes, shows that the two resilient classes, Consistent Resilience and Shock-to-Resilience, had comparably low likelihoods for both treatment-seeking and psychiatric diagnosis at the end of the study period. The highest likelihoods of both clinical outcomes were observed for the Strong Deterioration class, followed by the Mild Deterioration class, which also had a notable likelihood of reporting a psychiatric diagnosis and treatment seeking at the end of the 2-year study period. The Consistently High class showed intermediate rates of treatment-seeking and psychiatric diagnosis. These patterns comport with the substantive interpretation of these classes as clinically meaningful and distinct change profiles for depressive symptoms during the pandemic.

## 4. Discussion

This empirical investigation demonstrated how an overall view of symptom levels in the adult population masks key differences in depressive symptom change patterns during the pandemic. A Latent Change Score Mixture Model was implemented to approximate individual differences in depressive symptoms over time and found support for five different depressive symptom change patterns across a 24-month period during the pandemic. Consistent with previous literature from the first year of the pandemic [6, 7, 12], this study identified two subgroups that predominantly displayed resilience to adverse depressive symptomatology. One of these constituted nearly half the population, displaying consistently low levels of symptoms throughout the study period following a minor heightening in symptom levels during the first 3 months of the pandemic. The second subgroup of adults experienced an initial shock characterized by high levels of depressive symptoms at the onset of the pandemic before displaying adaptation and resilience within 3 months, which remained throughout the 2-year study period. Adults in the former consistent resilience group were more likely to have higher education, belong to the ethnic majority, live with others, and not have increased alcohol intake during the pandemic. Some of these characteristics (higher education, not living alone) were also protective factors of the shock-to-resilience group. The initial shock in depressive symptom levels distinguishing this second group from the consistently resilient group was related to increased financial and occupational worries at the onset of the pandemic and older age, with improvement in symptomatology related to more frequent engagement in physical activity. This is in line with previous findings identifying that financial assets protect against persistent depressive symptomatology during the present pandemic [40, 41]. The initial shock displayed by older aged individuals may have been related to greater infection fears, previously related to depressive symptomatology [42], and possibly explained by the greater risk of severe illness and mortality in these adults [43, 44]. These findings are also consistent with previous studies showing a positive association between physical activity and symptom reduction [45, 46], which highlights that this may be a useful strategy in mitigating adverse symptomatology in periods of lockdown and distancing.

Notably, the adults in the shock-to-resilience subgroup who displayed substantially heightened levels of depressive symptoms prior to recovery, reported highly frequent information seeking about the pandemic during its initial stages, consistent with previous findings linking greater pandemic news consumption to depressive symptomatology [47, 48]. This may be explained by a heightened stress response that can accompany exposure to negative and distressing news about a novel threat [49]. These individuals were also in a relationship, which may have been related to their recovery over time, given the identified associations between loneliness and depressive symptomatology during the earlier stages of the pandemic [50, 51]. Interestingly, having a preexisting psychiatric diagnosis was also related to this initial elevation and a reduction in depressive symptomatology during the first 3 months of the pandemic. This indicates that some individuals with preexisting mental health problems may have somewhat benefitted from the major contextual change and lockdown period occurring at the start of the pandemic. This interpretation is consistent with findings from another study, identifying that the increased time for self-care activities and the perception of lower external pressures following lockdown restrictions was, by some individuals, perceived as beneficial in processing mental health problems [52]. Together, the two predominantly resilient subgroups encompassed of approximately 55% of the population. Both groups were unlikely to report adverse future outcomes, including treatment seeking and reporting of a psychiatric diagnosis. This strengthens the message that the majority of the population displayed resilience to mental health adversities during the pandemic [6].

Corresponding to the known prevalence of depression in Norway [39], a subgroup encompassing approximately 9% of adults was identified, displaying consistently high depressive symptomatology during the pandemic period. Unsurprisingly, having a preexisting psychiatric diagnosis was among the most predictive variables of this subgroup. Preexisting mental health problems have also been associated with stronger increases in distress during the pandemic [53, 54], which may be sustained for particularly vulnerable groups. Individuals younger of age, single, living alone, binge drinking, and having financial and occupation concerns were also at greater risk of belonging to this consistently high group, which correspond to risk factors of sustained depressive states during pre-pandemic periods [55]. Greater engagement in physical activity protected against this depressive symptom change pattern, once again pointing at the possible mitigating utility of this ubiquitously available intervention during periods of infectious disease. The likelihood of this group reporting a future psychiatric diagnosis and seeking treatment was intermediate, which is meaningful as many of these individuals reported already having a psychiatric diagnosis at the onset of the study.

The two final subgroups of adults displayed deteriorating depressive symptom change patterns during the pandemic period, although to varying degrees and with large differences compared to their onset symptom levels. Combined, these groups encompassed approximately 35% of the adult population. Both subgroups displayed depressive symptomatology of minimal clinical relevance at the onset of the pandemic [22]. Key differences distinguished these two subgroups of adults from and following the third month of the pandemic. While one group displayed a slight increase in depressive symptom expression, the other group exhibited substantially higher deterioration during this period, an increase which was more than fivefold higher than the former group. Moreover, the strongly deteriorating group showed an increase in symptom severity of 12.1 points by the end of the 2-year period compared to its beginning, equivalent to a moderate-to-severe increase in depressive symptomatology [22]. These results indicate that the strongly deteriorating adults were quickly pushed toward a new and severe state of depression during the first 3 months of the pandemic, which has been maintained over time. This is consistent with patterns of deterioration in mental health observed in the United Kingdom during the early stages of the pandemic, where adverse gains in symptomatology accumulated during the first months of the pandemic [6, 12]. Both deleterious depressive symptom profiles were found to be detrimental beyond the adverse momentary experience of the symptoms in and of themselves, as these subgroups of adults displayed a high probability of reporting a psychiatric diagnosis and psychiatric treatment seeking by the end of the 2-year study period. Of note, both deteriorating subgroups of adults were identified above and beyond the consistently high subgroup displaying high depressive symptomatology from the onset of the pandemic. These findings are consistent with the novel global burden of disease study [56], which identified 53.2 million additional cases of major depressive disorder (i.e., an increase of 27.6%) during the pandemic. The strongly deteriorating subgroup had a higher probability to seek treatment at the end of the study period compared to the mildly deteriorating group of adults, which may be explained by the large differences in gained symptomatology between the groups across the pandemic period.

Several risk and protective factors relating to deterioration patterns were identified. Binge drinking and living alone at the onset of the pandemic were found to be strong predictors of both classes exhibiting deleterious depressive symptom change patterns. Extensive alcohol intake has been found to increase the risk of depression related to adverse neurophysiological and metabolic changes, in addition to disruptions in interpersonal functioning [57]. Living alone may further reduce the accessibility of social support, which has been found to be protective against depressive symptoms [58]. Older age and increased physical activity were protective features of the mildly deteriorating subgroup, with intensive information seeking during the onset of the pandemic further predictive of the change pattern identified within this subgroup. The protective link between physical activity and depression has been attributed to reduced inflammatory response and improved resilience to psychological stress [59, 60]. The adults in the strong deterioration subgroup were more likely to have lower education levels, and belonging to an ethnic minority was the only unique predictor of this subgroup. Both minority status and binge drinking substantially increased the odds of exhibiting this strongly deteriorating depressive symptom change pattern during the pandemic, where these adults shared alcohol consumption increase and lone residency as the most predictive characteristics of their group membership together with the consistently depressed individuals.

Importantly, having a preexisting psychiatric diagnosis was not predictive of the strongly deteriorating subgroup, which, together with the substantial gains in symptomatology during the pandemic and the finding of these adults reporting a high probability of treatment-seeking and obtainment of a psychiatric diagnosis at the end of the 2-year study period, indicates these adults may have transitioned from a predominantly asymptomatic to a disorder state during the pandemic [56]. These findings are consistent with reports of an added burden on the national healthcare system by the Norwegian Health Department, reporting additional increases in psychiatric treatment seeking among adults during the pandemic [61]. Should these trends continue, this increase in treatment-seeking (e.g., [61]) could emphasize a need for the development of scalable interventions (e.g., widely distributable internet-based treatments) to proactively alleviate the possible strain on mental health systems [62]. Unlike for all other subgroups, no actionable protective factors (e.g., physical activity) were identified for the strongly deteriorating subgroup of adults among the investigated variables, highlighting the need for future studies to identify routes to alleviate the adverse symptomatology of this newly emerged subgroup during the pandemic.

The present study has identified several key factors predicting differences in depressive symptom change patterns during the pandemic. Beyond these individual-level predictors, different contextual aspects accompanying the pandemic have been associated with mental health adversities in the literature. Two such factors include the societal infection rates and social containment policies (e.g., [56, 63]), with tentative findings suggesting that infection rates were most strongly tied to changes in anxiety symptoms [4, 64], while social containment policies were more strongly associated with depressive symptomatology [4]. More research is needed to examine whether specific components of the pandemic differentially impact different mental health outcomes across subgroups in the population.

### 4.1. Strengths and Limitations

This study has several noteworthy strengths, such as its large sample, nine repeated measurements over a 2-year period, adaptation of a modeling framework incorporating measurement error and enabling investigation of complex non-linear change patterns during the pandemic, and the use of validated measures with well-established clinical cutoffs. Importantly, the study extends the literature by mapping out the future adverse outcomes related to deleterious depressive symptom change patterns during the COVID-19 pandemic. This study also includes noteworthy limitations. While adults were randomly obtained and stratified to accurately represent population characteristics, the online procedure may have favored particular subgroups above others, such as older adults with more frequent computer usage. Efforts were taken to reduce such biases through additional recruitment on platforms more accessible through the elderly population, in addition to the employment of stratification procedures. While several key demographics (e.g., sex, education level, and presence of preexisting psychiatric diagnoses) were representative of the target population, the sample was younger and included fewer ethnic minorities than the population, serving as another limitation. Finally, the use of self-report measures is another limitation that precludes a more objective assessment of depressive symptomatology.

## 5. Future Directions

Several areas warrant further investigation that would be beneficial for the literature. In this study, large within-nation variability was found in depressive symptom change patterns during the pandemic. Future research investigating cross-national variability in mental health change profiles is needed, particularly in low- and middle-income countries, where the majority of the world population resides [65]. Leveraging the additional variability across nations with respect to different policy implementations and other contextual variables relevant during the pandemic, such internationally comparative investigations can provide additional opportunities for understanding the mechanistic processes underlying resilience and adverse patterns of change in mental health. Moreover, studying variability across different types of critical events is a key area for future preventive efforts. The extent to which processes and risk factors aggravating mental health identified during the present pandemic operate in a similar or different manner across other types of critical incidents (e.g., economic recessions, natural, and industrial disasters) is an important area for future preparedness, highlighting a need for prospective multiple incident studies in the literature.

## 6. Concluding Remarks

To conclude, the present findings have implications for mental health service planners and policymakers. The identified window of sensitivity for depressive adversities calls for increased vigilance of psychiatric symptoms during the first 3 months of pandemic periods and a target point for insertion of preventive measures, after which symptom transitions stabilize and are less subjectable to change throughout the pandemic period. As periods of infectious disease may follow similar behavioral mitigation strategies and be subject to similar psychological mechanisms (i.e., need for information obtainment), it is likely that these findings can help inform future pandemic preparedness. Among modifiable possibilities, dissemination about the adverse associations tied to intensive information-seeking behavior and the beneficial impact of physical activity during periods of reduced mobility and isolation may be a fruitful strategy. This study further highlights the role of financial and occupational worries in aggravating depressive symptoms, suggesting that socioeconomic policies may be of importance in post-pandemic recovery programs and as preventive measures in future pandemics during phases of additional economic vulnerability (e.g., lockdown periods). Echoing previous studies [6] and consistent with ongoing national and global reports [56, 61], the findings highlight that mental health services may expect to see an increase in referrals, necessitating careful considerations and logistical planning by health and government agencies.

## Figures and Tables

**Table 1:** Demographic information of the participants.

Subgroups	*N* (%)
All	4,361 (100%)
Sex	
Female	2,152 (49.34%)
Male	2,183 (50.06%)
Missing	26 (0.60%)
Age, years (*M* = 37.48, *SD* = 14.81)	
18–30	1,983 (45.47%)
31–44	1,108 (25.41%)
45–64	1,037 (23.78%)
65+	233 (5.34%)
Education level	
Compulsory school	522 (11.97%)
Upper secondary high school	1,786 (40.95%)
Currently studying	510 (11.70%)
Any university degree	1,543 (35.38%)
Relationship status	
Single or divorced	1,765 (40.47%)
In a relationship	2,596 (59.53%)
Ethnic status	
Nonminority	4,136 (94.84%)
Ethnic minority	225 (5.16%)
Preexisting psychiatric diagnosis	
Yes	850 (19.49%)
No	3,511 (80.51%)

**Table 2:** Results of the latent change score mixture model (LCSMM).

	Estimate	SE	*z*	*p*
Class 1: Strong Deterioration (*N* = 295; 6.77%)				
Means				
* η*_t1_	5.22	0.82	6.40	<0.001*⁣*^*∗*^
*δη*_t2_	13.07	0.75	17.45	<0.001*⁣*^*∗*^
*δη*_t3_	0.73	0.69	1.05	0.294
*δη*_t4_	1.41	0.54	2.63	0.009*⁣*^*∗*^
*δη*_t5_	−1.11	0.49	−2.26	0.024*⁣*^*∗*^
*δη*_t6_	−1.08	0.65	−1.67	0.096
*δη*_t7_	0.29	0.94	0.31	0.758
*δη*_t8_	1.26	0.72	1.76	0.079
*δη*_t9_	−1.43	0.81	−1.77	0.076
Class 2: Consistently High (*N* = 371; 8.50%)				
Means				
* η*_t1_	14.36	1.92	7.47	<0.001*⁣*^*∗*^
*δη*_t2_	−0.13	0.98	−0.13	0.897
*δη*_t3_	1.85	0.65	2.83	0.005*⁣*^*∗*^
*δη*_t4_	−0.34	0.47	−0.71	0.475
*δη*_t5_	−0.27	0.52	−0.52	0.601
*δη*_t6_	−2.23	0.56	−3.96	<0.001*⁣*^*∗*^
*δη*_t7_	0.37	0.72	0.51	0.612
*δη*_t8_	0.76	0.82	0.93	0.354
*δη*_t9_	−0.81	0.68	−1.18	0.237
Class 3: Mild Deterioration (*N* = 1,267; 29.04%)				
Means				
* η*_t1_	5.99	0.51	11.76	<0.001*⁣*^*∗*^
*δη*_t2_	2.43	0.33	7.39	<0.001*⁣*^*∗*^
*δη*_t3_	1.57	0.23	6.90	<0.001*⁣*^*∗*^
*δη*_t4_	0.02	0.20	0.08	0.935
*δη*_t5_	−0.26	0.22	−1.22	0.224
*δη*_t6_	−1.56	0.25	−6.14	<0.001*⁣*^*∗*^
*δη*_t7_	0.39	0.29	1.33	0.183
*δη*_t8_	0.88	0.30	2.92	0.003*⁣*^*∗*^
*δη*_t9_	−0.70	0.29	−2.45	0.014*⁣*^*∗*^
Class 4: Shock-to-Resilience (*N* = 574; 13.17%)				
Means				
* η*_t1_	17.33	0.38	46.05	<0.001*⁣*^*∗*^
*δη*_t2_	−13.50	0.48	−28.32	<0.001*⁣*^*∗*^
*δη*_t3_	1.43	0.23	6.18	<0.001*⁣*^*∗*^
*δη*_t4_	0.10	0.23	0.46	0.649
*δη*_t5_	−0.24	0.25	−0.96	0.337
*δη*_t6_	−1.25	0.25	−5.07	<0.001*⁣*^*∗*^
*δη*_t7_	0.25	0.27	0.93	0.353
*δη*_t8_	0.14	0.28	0.51	0.609
*δη*_t9_	−0.11	0.29	−0.39	0.694
Class 5: Consistent Resilience (*N* = 1,854; 42.52%)				
Means				
* η*_t1_	4.76	0.13	37.46	<0.001*⁣*^*∗*^
*δη*_t2_	−1.67	0.22	−7.75	<0.001*⁣*^*∗*^
*δη*_t3_	0.58	0.10	6.10	<0.001*⁣*^*∗*^
*δη*_t4_	0.08	0.09	0.85	0.394
*δη*_t5_	−0.23	0.10	−2.30	0.022*⁣*^*∗*^
*δη*_t6_	−0.58	0.11	−5.19	<0.001*⁣*^*∗*^
*δη*_t7_	0.01	0.11	0.06	0.954
*δη*_t8_	0.40	0.12	3.24	0.001*⁣*^*∗*^
*δη*_t9_	−0.27	0.14	−1.99	0.046*⁣*^*∗*^

*Note. η*_t1_ = latent intercept at T1 (March 2020); *δη*_t2_ = latent change from T1 to T2 (March to July, 2020); *δη*_t3_ = latent change from T2 to T3 (July to December, 2020); *δη*_t4_ = latent change from T3 to T4 (December 2020 to February, 2021); *δη*_t5_ = latent change from T4 to T5 (February to May, 2021); *δη*_t6_ = latent change from T5 to T6 (May to August, 2021)); *δη*_t7_ = latent change from T6 to T7 (August to November, 2021); *δη*_t8_ = latent change from T7 to T8 (November, 2021 to January, 2022); *δη*_t9_ = latent change from T8 to T9 (January to March, 2022). *⁣*^*∗*^*p* < 0.05.

**Table 3:** Odds ratios (OR) for the different predictors of class membership relative to the reference (Consistent Resilience) class, along with the 95% confidence intervals of ORs.

Predictor	Class	OR	Lower CI	Upper CI	*p*
Age	1 (SD)	0.93	0.70	1.22	0.593
	2 (CH)	0.43	0.29	0.65	<0.001*⁣*^*∗*^
	3 (MD)	0.69	0.56	0.84	<0.001*⁣*^*∗*^
	4 (SR)	0.45	0.34	0.60	<0.001*⁣*^*∗*^

Living alone	1 (SD)	2.98	1.65	5.40	<0.001*⁣*^*∗*^
	2 (CH)	3.13	1.52	6.44	0.002*⁣*^*∗*^
	3 (MD)	2.36	1.46	3.82	<0.001*⁣*^*∗*^
	4 (SR)	2.03	1.14	3.59	0.015*⁣*^*∗*^

Relationship	1 (SD)	1.02	0.58	1.81	0.936
	2 (CH)	0.51	0.27	0.97	0.040*⁣*^*∗*^
	3 (MD)	0.98	0.65	1.48	0.929
	4 (SR)	0.52	0.32	0.83	0.006*⁣*^*∗*^

Education	1 (SD)	0.78	0.60	0.99	0.044*⁣*^*∗*^
	2 (CH)	0.81	0.58	1.12	0.199
	3 (MD)	0.89	0.74	1.07	0.203
	4 (SR)	0.73	0.58	0.92	0.009*⁣*^*∗*^

Information seeking	1 (SD)	1.12	0.86	1.46	0.403
	2 (CH)	1.16	0.83	1.62	0.393
	3 (MD)	1.22	1.01	1.47	0.036*⁣*^*∗*^
	4 (SR)	1.30	1.06	1.56	0.013*⁣*^*∗*^

Ethnic minority	1 (SD)	3.66	1.36	9.81	0.010*⁣*^*∗*^
	2 (CH)	2.59	0.56	11.93	0.223
	3 (MD)	2.30	0.95	5.54	0.064
	4 (SR)	1.11	0.36	3.43	0.855

Binge drinking	1 (SD)	16.78	2.86	98.52	0.002*⁣*^*∗*^
	2 (CH)	13.99	2.28	85.92	0.004*⁣*^*∗*^
	3 (MD)	6.84	1.17	39.88	0.033*⁣*^*∗*^
	4 (SR)	1.76	0.22	13.89	0.590

Physical activity	1 (SD)	0.88	0.73	1.06	0.183
	2 (CH)	0.59	0.46	0.77	<0.001*⁣*^*∗*^
	3 (MD)	0.84	0.73	0.97	0.018*⁣*^*∗*^
	4 (SR)	0.67	0.57	0.79	<0.001*⁣*^*∗*^

Psychiatric diagnosis	1 (SD)	1.03	0.45	2.34	0.944
	2 (CH)	12.21	6.02	24.76	<0.001*⁣*^*∗*^
	3 (MD)	1.42	0.79	2.55	0.237
	4 (SR)	11.35	6.57	19.61	<0.001*⁣*^*∗*^

Sex	1 (SD)	1.00	0.58	1.73	0.995
	2 (CH)	0.58	0.30	1.12	0.106
	3 (MD)	0.80	0.53	1.20	0.279
	4 (SR)	0.68	0.42	1.10	0.113

Worry about job and economy	1 (SD)	1.03	0.86	1.24	0.748
	2 (CH)	1.51	1.26	1.80	<0.001*⁣*^*∗*^
	3 (MD)	1.07	0.96	1.20	0.237
	4 (SR)	1.53	1.35	1.74	<0.001*⁣*^*∗*^

*Note*. *⁣*^*∗*^ *p* <  0.05. CH, Consistently High (Class 2); MD, Mild Deterioration (Class 3); SD, Strong Deterioration (Class 1); SR, Shock-to-Resilience (Class 4).

## Data Availability

Our received ethical approval from the Norwegian Centre for Research Data (NSD) and The Regional Committee for Medical and Health Research Ethics (REK) precludes submission of raw data to public repositories. Access to the data can be granted from the principal investigator Omid V. Ebrahimi following ethical approval of a suggested project plan for the use of data granted by NSD and REK.

## Additional Points

*Transparency and Openness*. In accordance with the Transparency and Openness Promotion (TOP) guidelines, all used methods in this study are detailed and cited in the article. These analytical strategies are further detailed in the supplementary materials, with the code available on request from the corresponding author.

## Supplementary Materials

Supplementary 1 Additional details about the class enumeration procedure and demographic information of the participants across all nine waves of the study. Table S1: clustering criteria and information criteria per class derived from the latent change score mixture model. Table S2: demographic information of the participants across all nine waves of the study.

Supplementary 2 Figure 1: population-level change patterns in depressive symptoms across the pandemic period.

Supplementary 3 Figure 2: scores of depressive symptom severity (PHQ-9) for a random subset of 100 individuals with posterior probabilities ≥0.9 of belonging to each of the five profiles of depressive symptom change patterns during the COVID-19 pandemic.
