# Supplementary material for: Depressive Symptom Change Patterns during the COVID-19 Pandemic and Their Impact on Psychiatric Treatment Seeking: A 24-Month Observational Study of the Adult Population
Source: Depress Anxiety. 2024 Aug 5;2024:1272738. doi: 10.1155/2024/1272738 (PMC11918502; doi:10.1155/2024/1272738)
Supplement: Supplementary 1 — Additional details about the class enumeration procedure and demographic information of the participants across all nine waves of the study. Table S1: clustering criteria and information criteria per class derived from the latent change score mixture model. Table S2: demographic information of the participants across all nine waves of the study. [file 1272738.f1.pdf]

Depressive symptom change patterns during the COVID-19 pandemic and their impact on psychiatric treatment seeking: A 24-month observational study of the adult population

Supplementary Appendix and Supplementary Tables S1 and S2

Class Enumeration

Table S1 below presents the model performance metrics for each of the eight models tested. Overall, as described in the Results section, the preponderance of our class selection criteria (cf. Methods section) favored a five-class solution, which was further revealed as a robust and stable solution consistent with the literature. As expected, given the large sample size of the study and its high power to detect additional classes based on trivial changes in mean levels, information criteria continued to decrease upon addition of more classes. These decreases however, portrayed diminished returns following five classes and this, coupled with increasing model instability, argued for selection of no more than five classes (Nylund-Gibson & Choi, 2018; Sinha et al., 2021).

Additional sensitivity analyses were conducted comparing these quantitative results to theory and previous empirical findings which further supported the selection of a five-class model. First, the five-class solution was found to correspond with previously identified number of classes in the literature during the early stages of the pandemic, investigating mental health up to October 2020 (Pierce et al., 2021). Second, we compared the final five-class solution to the four-class model, the latter of which failed to retrieve the subgroup of individuals with pre-existing and chronic depressive problems (cf. Consistently High class; Figure 1 in the manuscript). The five-class model estimates this group to comprise 8.5% of the population, a figure that is closely corroborated by the Norwegian Institute of Public

Health (2016) estimate that 10% of the population of Norway suffered from depressive problems pre-pandemic. Finally, when introducing the six-class model and additional models, no novel unique patterns were retrieved, with additional classes revealing unstable results (cf. replication of loglikelihood values) and further splitting up previously identified classes (i.e., two similar pairs of the identified Mild Deterioration class; Figure 1 in the manuscript) into more granular subgroup with solely minimal differences in mean levels, further suggesting that the principal patterns in the data were successfully retrieved with the five-class model.

Table S1. Clustering Criteria and Information Criteria per class derived from the Latent Change Score Mixture Model.

**Classes**	**BIC**	**AIC**	**CAIC**	**ssBIC**	**Entropy**	**P-BLRT**	**Class proportion (%)**
**1**[a]	112837.49	112773.68	112847.49	112805.71	-	-	100
**2**[a]	107049.66	106922.05	107069.66	106986.11	0.68	17331.50*	74.42, 25.58
**3**[b]	105549.80	105358.39	105579.80	105454.47	0.66	1583.66*	57.06, 29.71, 13.23
**4**[a]	103646.37	103391.15	103686.37	103519.26	0.69	1987.24*	45.37, 13.81, 12.12, 28.70
**5**[a]	103012.21	102693.19	103062.21	102853.34	0.71	717.96*	8.50, 29.04, 6.77, 13.17, 42.52
**6**[b]	102550.82	102167.99	102610.82	102360.16	0.66	545.20*	4.90, 16.21, 25.67, 33.45, 5.98, 13.79
**7**[b]	102209.96	101763.32	102279.96	101987.52	0.67	424.67*	5.01, 31.01, 6.27, 14.83, 5.44, 26.26, 11.18
**8**[b]	101981.49	101471.06	102061.49	101727.29	0.67	312.27*	10.67, 29.71, 14.62, 5.40, 1.32, 25.94, 6.19, 6.15

Note. [a] Best loglikelihood consistently replicated; [b] Best loglikelihood did not consistently replicate; BIC: Bayesian Information Criterion; AIC: Akaike Information Criterion; CAIC: Consistent Akaike Information Criterion; ssBIC: sample size adjusted Bayesian Information Criterion; P-BLRT: Parametric Bootstrapped Likelihood Ratio Test. * $p < .05$.

Supplementary Table S2. Demographic information of the participants across all nine waves of the study.

**Subgroups**	**T1** ***N*** **(%)**	**T2** ***N*** **(%)**	**T3** ***N*** **(%)**	**T4** ***N*** **(%)**	**T5** ***N*** **(%)**	**T6** ***N*** **(%)**	**T7** ***N*** **(%)**	**T8** ***N*** **(%)**	**T9** ***N*** **(%)**
**All**	4361 (100%)	2158 (100%)	2239 (100%)	1963 (100%)	1811 (100%)	1405 (100%)	1426 (100%)	1110 (100%)	1269 (100%)
**Sex**									
Female	2152 (49.34%)	1023 (47.40%)	1082 (48.33%)	952 (48.50%)	855 (47.21%)	634 (45.12%)	672 (47.13%)	534 (48.11%)	593 (46.57%)
Male	2183 (50.06%)	1118 (51.81%)	1144 (51.09%)	999 (50.89%)	949 (52.40%)	762 (54.24%)	747 (52.38%)	573 (51.44%)	672 (52.96%)
Missing	26 (0.60%)	17 (0.79%)	13 (0.58%)	12 (0.61%)	7 (0.39%)	9 (0.64%)	7 (0.49%)	5 (0.45%)	6 (0.47%)
**Age, years**	*M* = 37.48, *SD* = 14.81	*M* = 38.56, *SD* = 14.96	*M* = 38.51, *SD* = 15.00	*M* = 38.72, *SD* = 15.03	*M* = 38.91, *SD* = 15.18	*M* = 39.71, *SD* = 15.16	*M* = 38.98, *SD* = 15.41	*M* = 38.89, *SD* = 15.31	*M* = 38.99, *SD* = 15.38
18-30	1983 (45.47%)	910 (42.17%)	944 (42.16%)	815 (41.52%)	757 (41.80%)	558 (39.72%)	605 (42.43%)	471 (42.43%)	547 (43.11%)
31-44	1108 (25.41%)	572 (26.51%)	593 (26.49%)	527 (26.85%)	464 (25.62%)	367 (26.12%)	350 (24.54%)	279 (25.41%)	307 (24.19%)
45-64	1037 (23.78%)	549 (25.44%)	569 (25.41%)	500 (25.47%)	474 (26.17%)	383 (27.26%)	370 (25.95%)	282 (25.41%)	324 (25.53%)
65+	233 (5.34%)	127 (5.88%)	133 (5.94%)	121 (6.16%)	116 (6.41%)	97 (6.90%)	101 (7.08%)	78 (7.03%)	91 (7.17%)
**Education level**									
Compulsory School	522 (11.97%)	239 (11.08%)	253 (11.30%)	227 (11.57%)	192 (10.60%)	156 (11.10%)	169 (11.85%)	129 (11.62%)	140 (11.03%)
Upper secondary high school	1786 (40.95%)	873 (40.45%)	908 (40.55%)	807 (41.11%)	739 (40.81%)	557 (39.65%)	575 (40.32%)	460 (41.44%)	511 (40.27%)
Currently studying	510 (11.70%)	249 (11.54%)	263 (11.75%)	225 (11.46%)	210 (11.60%)	152 (10.82%)	163 (11.43%)	128 (11.53%)	141 (11.11%)

Any university degree	1543 (35.38%)	797 (36.93%)	815 (36.40%)	704 (35.86%)	670 (36.99%)	540 (38.43%)	519 (36.40%)	393 (35.41%)	477 (37.59%)
**Relationship status**									
Single or divorced	1765 (40.47%)	866 (40.13%)	913 (40.78%)	790 (40.24%)	713 (39.37%)	537 (38.22%)	560 (39.27%)	427 (38.46%)	500 (39.40%)
In a relationship	2596 (59.53%)	1292 (59.87%)	1326 (59.22%)	1173 (59.76%)	1098 (60.63%)	868 (61.77%)	866 (60.73%)	683 (61.53%)	769 (60.60%)
**Ethnic status**									
Non-minority	4136 (94.84%)	2047 (94.86%)	2127 (95.00%)	1859 (94.70%)	1713 (94.59%)	1332 (94.80%)	1353 (94.88%)	1064 (95.86%)	1202 (94.72%)
Ethnic minority	225 (5.16%)	111 (5.14%)	112 (5.00%)	104 (5.29%)	98 (5.41%)	73 (5.20%)	73 (5.12%)	46 (4.14%)	67 (5.28%)
**Preexisting psychiatric diagnosis**									
Yes	850 (19.49%)	444 (20.57%)	467 (20.86%)	399 (20.33%)	355 (19.60%)	264 (18.79%)	260 (18.23%)	219 (19.73%)	253 (19.94%)
No	3511 (80.51%)	1714 (79.43%)	1772 (79.14%)	1564 (79.67%)	1456 (80.40%)	1141 (81.21%)	1166 (81.77%)	891 (80.27%)	1016 (80.06%)

References

Norwegian Institute of Public Health. (2016). Mental illness among adults in Norway. In: Public Health Reports – Health Status in Norway. https://www.fhi.no/en/op/hin/mental-health/psykisk-helse-hos-voksne/.

Nylund-Gibson, K., & Choi, A. Y. (2018). Ten frequently asked questions about latent class analysis. *Translational Issues in Psychological Science, 4(*4), 440. doi:10.1037/tps0000176

Pierce, M., Hope, H., Ford, T., Hatch, S., Hotopf, M., John, A., Kontopantelis, E., Webb, R., Wessely, S., McManus, S., & Abel, K. M. (2020). Mental health before and during the COVID-19 pandemic: A longitudinal probability sample survey of the UK population. *The Lancet Psychiatry*, *7*(10), 883–892. https://doi.org/10.1016/S2215-0366(20)30308-4

Sinha, P., Calfee, C. S., & Delucchi, K. L. (2021). Practitioner's guide to latent class analysis: methodological considerations and common pitfalls. *Critical care medicine*, *49*(1), e63.
