# Supplementary figures and images for: Depressive Symptom Change Patterns during the COVID-19 Pandemic and Their Impact on Psychiatric Treatment Seeking: A 24-Month Observational Study of the Adult Population
Source: Depress Anxiety. 2024 Aug 5;2024:1272738. doi: 10.1155/2024/1272738 (PMC11918502; doi:10.1155/2024/1272738)

Figure S1. Population-level change patterns in depressive symptoms across the pandemic period.

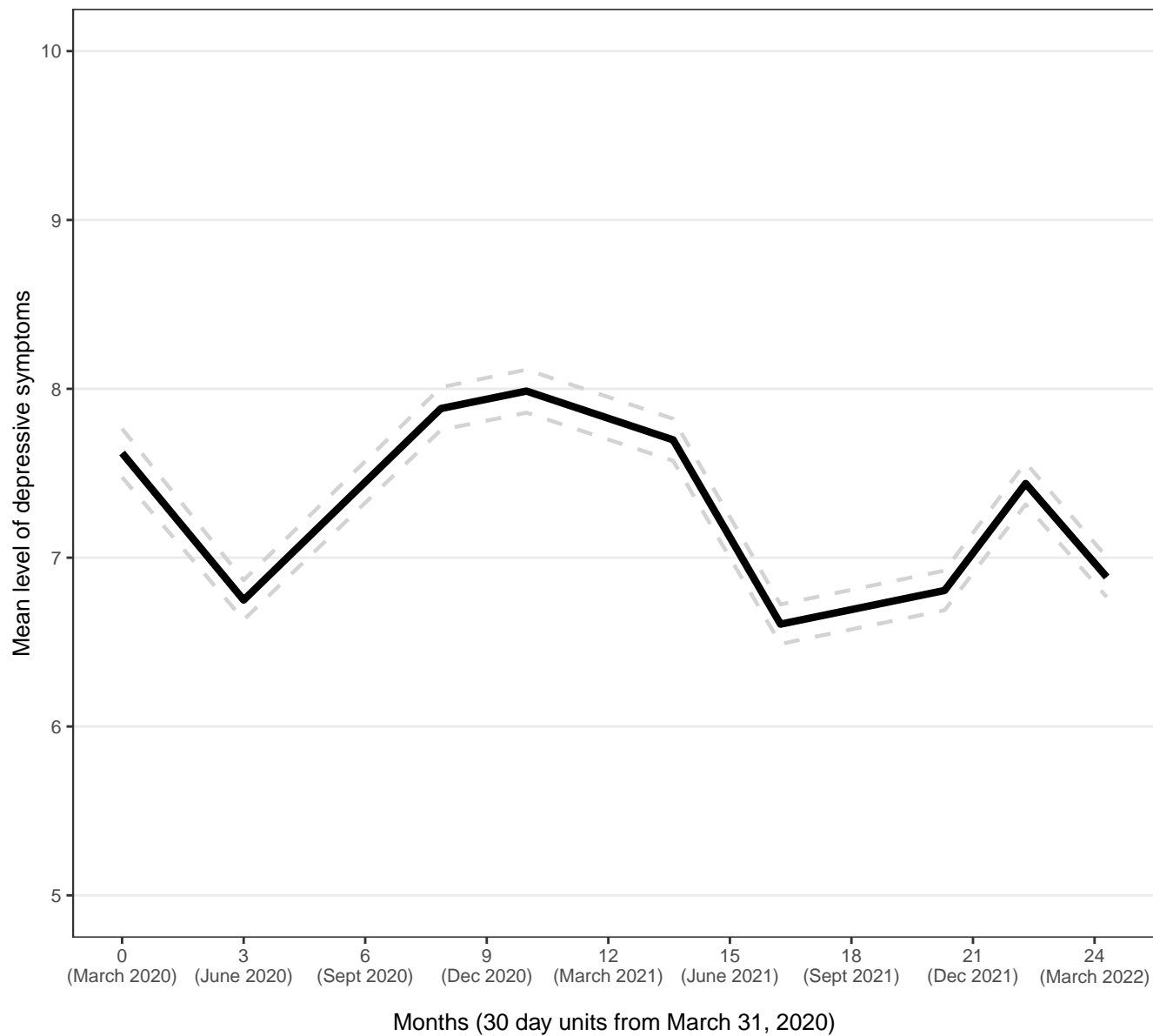

Supplement: Supplementary 2 — Figure 1: population-level change patterns in depressive symptoms across the pandemic period. [file 1272738.f2.pdf]
