# Supplementary material for: Depressive Symptom Change Patterns during the COVID-19 Pandemic and Their Impact on Psychiatric Treatment Seeking: A 24-Month Observational Study of the Adult Population
Source: Depress Anxiety. 2024 Aug 5;2024:1272738. doi: 10.1155/2024/1272738 (PMC11918502; doi:10.1155/2024/1272738)

Figure S2

Scores of Depressive Symptom Severity (PHQ-9) for a Random Subset of 100 Individuals With Posterior Probabilities ≥ .9 of Belonging to Each of the Five Profiles of Depressive Symptom Change Patterns During the COVID-19 Pandemic

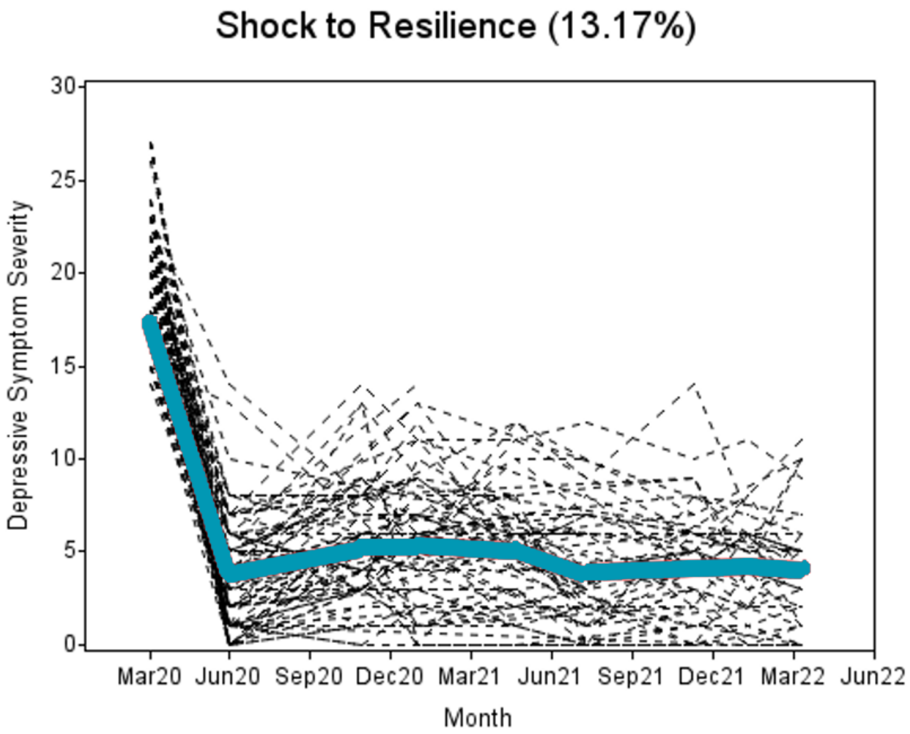

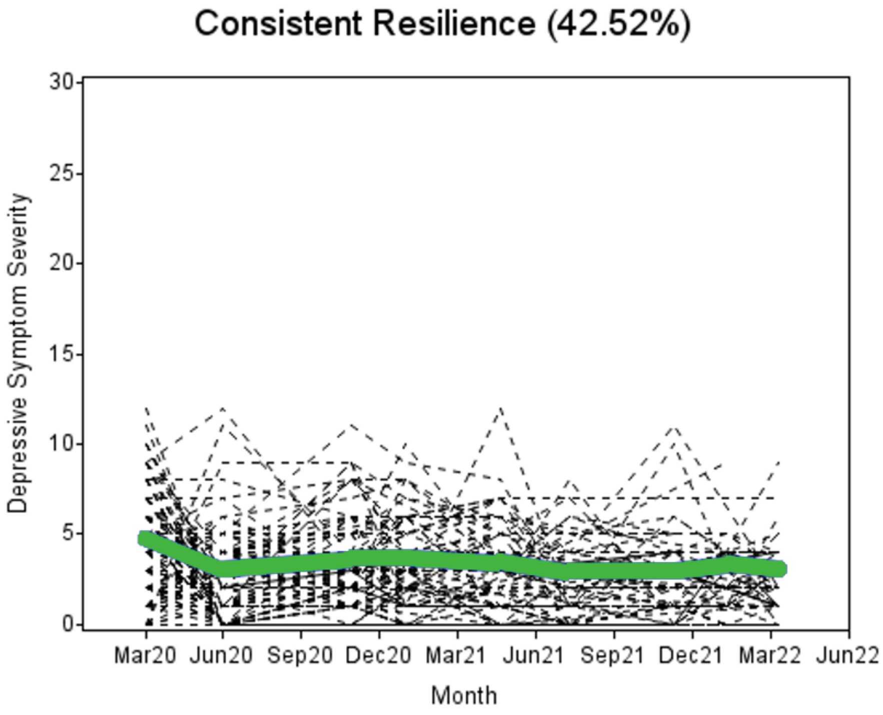

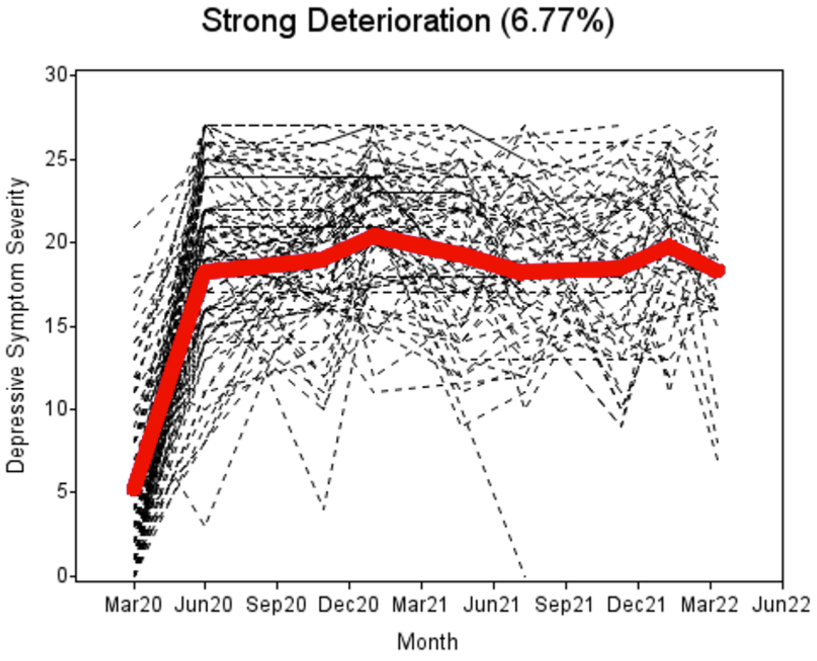
Strong Deterioration (6.77%)
Depressive Symptom Severity
0
5
10
15
20
25
30
Mar20 Jun20 Sep20 Dec20 Mar21 Jun21 Sep21 Dec21 Mar22 Jun22
Month

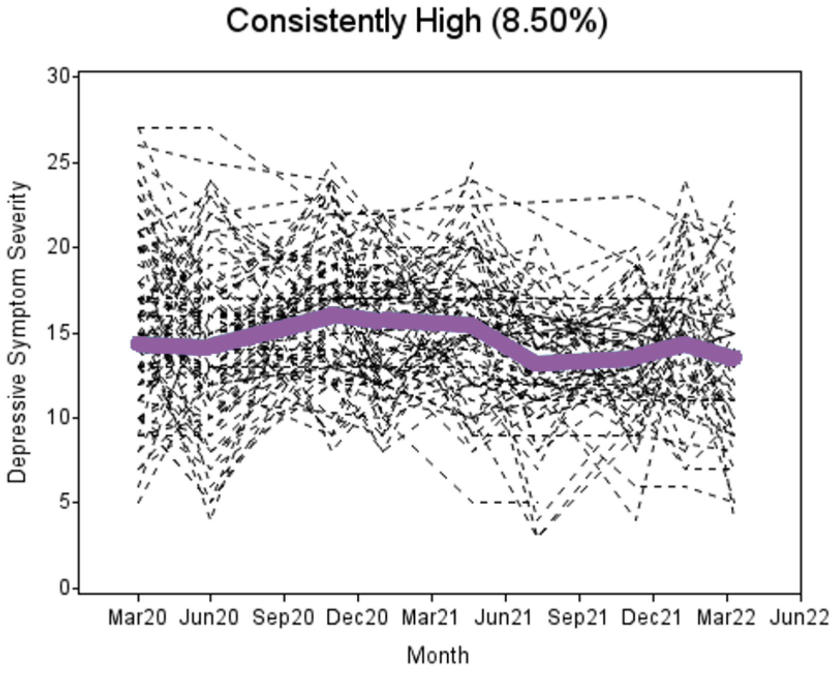
Consistently High (8.50%)
Depressive Symptom Severity
0
5
10
15
20
25
30
Mar20 Jun20 Sep20 Dec20 Mar21 Jun21 Sep21 Dec21 Mar22 Jun22
Month

Mild Deterioration (29.04%)

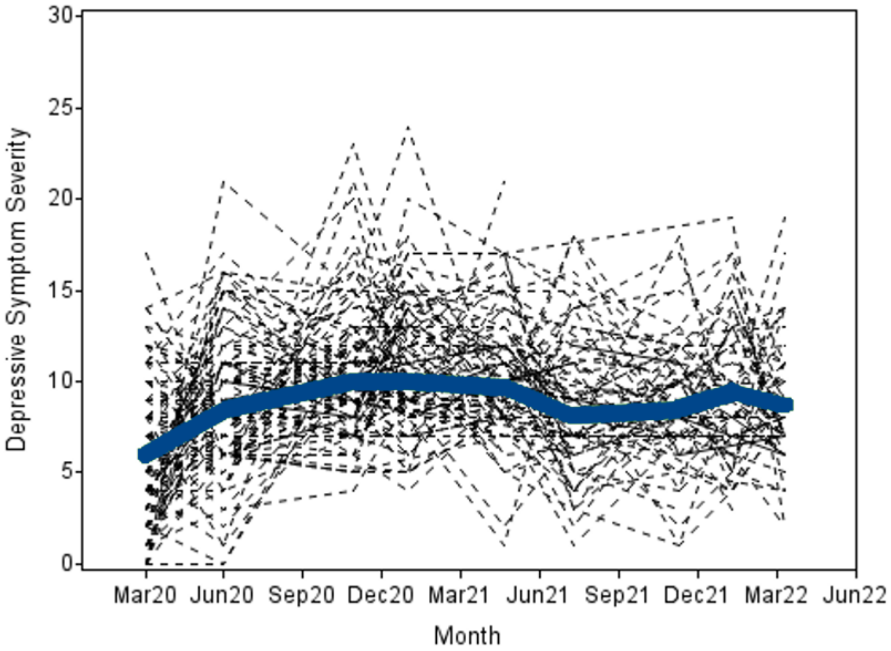

Supplement: Supplementary 3 — Figure 2: scores of depressive symptom severity (PHQ-9) for a random subset of 100 individuals with posterior probabilities ≥0.9 of belonging to each of the five profiles of depressive symptom change patterns during the COVID-19 pandemic. [file 1272738.f3.pdf]
